# Differential effects of pre and post-payment on neurologists' response rates to a postal survey

**DOI:** 10.1186/1471-2377-10-100

**Published:** 2010-10-25

**Authors:** Richard AA Kanaan, Simon C Wessely, David Armstrong

**Affiliations:** 1 Department of Psychological Medicine, Institute of Psychiatry, King's College London, London, UK; 2 Department of General Practice, King's College London, London, UK

## Abstract

**Background:**

Monetary incentives are an effective way of increasing response rates to surveys, though they are generally less effective in physicians, and are more effective when the incentive is paid up-front rather than when made conditional on completion.

**Methods:**

In this study we examine the effectiveness of pre- and post-completion incentives on the response rates of all the neurologists in the UK to a survey about conversion disorder, using a cluster randomised controlled design. A postal survey was sent to all practicing consultant neurologists, in two rounds, including either a book token, the promise of a book token, or nothing at all.

**Results:**

Three hundred and fifty-one of 591 eligible neurologists completed the survey, for a response rate of 59%. While the post-completion incentive exerted no discernible influence on response rates, a pre-completion incentive did, with an odds-ratio of 2.1 (95% confidence interval 1.5 - 3.0).

**Conclusions:**

We conclude that neurologists, in the UK at least, may be influenced to respond to a postal survey by a pre-payment incentive but are unaffected by a promised reward.

## Background

Among strategies for improving survey response rates, the use of a monetary incentive is one of the most effective - more than doubling the odds of a response [1]. These effects are less well studied in physicians [2], but what evidence there is suggests those odds are somewhat reduced though the same principles apply: larger incentives are more effective, and pre-paying the incentive is more effective than promising a payment on completion [1,3,4]. The effect of post-payment has rarely been studied directly, however, and the differential effects of pre- and post-payment of the incentive seem to vary by medical specialty [5,6]. This variation would suggest specialties vary in their susceptibility to the different processes that are thought to make these incentives effective. These effects have never been specifically studied in neurologists, to our knowledge. This study was designed to examine the effects of pre and post-payment of an incentive on a postal survey of the neurologists working in the United Kingdom (UK).

## Methods

A postal survey was sent to all practicing consultant neurologists of the Association of British Neurologists (ABN) in February 2009, using a cluster randomised controlled design. Prior to sending out the survey, the neurologists were grouped by their hospital or other working location and these location-groups were randomly assigned to three incentive conditions by a statistician blinded to the nature of the groups - a group being assigned to each incentive condition with equal probability, and independently of the assignment of the other groups (division by working location was to prevent revelation of the differential incentives between the neurologists from influencing their responses). The first incentive group were sent a £10 (~ $16) book token; the second group were told they would receive a £10 book token on returning the survey; the third group received no mention of any incentive. After approximately one month, a second survey was sent to all those who had not responded to the first round. This survey had identical questions to the first, was the same for all three groups, and made no mention of any incentive. Both rounds were sent using printed postage, and included a return envelope with a first-class stamp. The survey contained 33 questions, largely multiple-choice but with some free text, first covering demographics and details of the clinician's practice, and then addressing their understanding and management of conversion disorder, a condition that many would see as outside of their remit but one that greatly challenges them nonetheless. It extended to four pages, including instructions. Data from the survey were entered into and analysed with SPSS 16.0 (SPSS Inc, Illinois). The survey was approved by a local ethical review board; response to the survey was taken to indicate consent by the neurologists.

## Results

The ABN supplied a list of 634 names and addresses. Thirteen of these were overseas and five were known by the authors to be non-neurologists (one of the authors of this paper, for example) leaving 616 names. Three hundred and nineteen (52%) responded to the first round, and a further 59 responded to the second round, for an aggregate response of 378 (62%) plus five that were returned 'addressee unknown'. Twenty-seven of these were not included in the analysis, however, either because they did not fill in the survey (7 subjects) or because they gave us reason to exclude their responses. Reasons were being a neurophysiologist (8 subjects) or other non-neurologist (5 subjects); or being retired (3 subjects), on long-term sick leave (1 subject) or otherwise not seeing patients (3 subjects). There were no differences in the number of those 'giving reasons' between the three incentive groups. Excluding them gave an adjusted completion rate of 351 from 591 eligible subjects (59%, Table 1).

**Table 1:** Response rates for Incentives

Incentive	First Round (300 replies)	Second Round (51 replies)	Aggregate (351 replies, 59%)
Token Sent (n = 214)	131/214 (61%)	20/83 (24%)	151/214 (71%)

Token Promised (n = 147)	71/147 (48%)	8/76 (11%)	79/147 (54%)

No Token (n = 230)	98/230 (43%)	23/132 (17%)	121/230 (53%)

Difference	p = 0.0004	p = 0.08	p = 0.0002

The difference in the response rate was highly significant between the three groups (χ2 = 15.8, df = 2, p = 0.0004), with the group sent the incentive responding 61% of the time, compared with 48% for those promised the incentive, and 43% for those without incentive (see Table 1). This difference was sustained into the second round, even though the incentive was only actually sent/promised in the first round, though the difference did not reach significance (χ2 = 5.1, df = 2, p = 0.08), but heightened the aggregate significance over both rounds (χ2 = 17.4, df = 2, p = 0.0002). There were no significant differences in response rates between the token-promised and no-token groups at either the first (p = 0.3) or second rounds (p = 0.3), or their aggregate (p = 0.9). Comparing the aggregate of those sent tokens to the other two groups gave a final odds-ratio of 2.1 (.95 CI 1.5-3.0) for the effect of being sent a book token incentive.

There were no differences between the groups in terms of their demographic or practice characteristics as reported in the survey. Using late respondents (respondents to the second round) as proxies for non-respondents (a standard method for assessing survey representativeness [7]) found no differences in demographic or practice characteristics, suggesting that the survey respondents as a whole were not themselves biased in such terms. In terms of effects on response to the survey questions themselves, the groups differed on response to two items at an uncorrected threshold of p < 0.05, though the significance of the differences would not have survived any correction for multiple comparisons. One of the items was a diagnostic response to a clinical vignette, (χ2 = 12.7, df = 4, p = 0.013), the other was willingness to copy clinic letters to clients (χ2 = 11.9, df = 2, p = 0.003).

## Discussion

This is the first study we know of that directly examines the effects of pre-and post- payment vs. no payment in physicians, and the first to do so amongst neurologists. The results suggest that there is markedly differential effectiveness of social-psychological influences in this group: 'greed' doesn't work, but 'guilt' does.

Pre-payment incentives derive their effectiveness from 'guilt', or what social psychologists refer to as 'the norm of reciprocity' [8]. When the unsolicited book-token is received, there is no further (monetary) benefit to replying [9]; but there is a social-psychological norm that the recipient should respond: one kindness deserves another. In this condition, being sent a reminder letter would serve to reinforce the 'guilt' that the neurologist may be expected to feel by not having responded despite having kept the book token: this may account for the continued increased response in the pre-payment group to the second mailing, even though there was no incentive included in that mailing.

Post-payments, by contrast, impose little social obligation on the subject, but demonstrate a respect for the subject's time that may in itself be considered a favour to be returned, and - if large enough - may induce a desire on the subject's part for the promised reward [10]. Ten pounds sterling is not a great deal (about enough to buy one new paperback book), but it compares favourably with the rate of pay a consultant might receive from the UK National Health Service for the approximately ten minutes the survey should have taken to fill in (though considerably less than they might expect to receive from additional, private work). It may well be that the neurologists would have responded more enthusiastically to a larger post-payment, but at this level of incentive the neurologists showed no evidence that they were influenced by 'greed'. Indeed, of those sent the book token in advance, three returned it (one who had completed the survey and two who had not), and of those promised the book token, two wrote on the survey that they did not want it and a third that it be sent to charity. Unsurprisingly, there was no increased response to the second round amongst those promised the token: those who had been unmoved by the offer the first time round could not expect to be inspired by whatever vague recollection they might have of the offer some weeks later. It may seem that the effects of 'greed', while small, were only non-significant because of the sample size. While that may possibly be true, the sample for that incentive arose as a result of random allocation over a complete sample of the neurologists in the UK, so could not have been expanded.

How broadly these findings may be generalised is uncertain. Response to incentives has been shown to vary with specialty [5], and it may be that neurologists are particularly subject to 'guilt', or particularly immune to 'greed'. It may also be that these findings apply particularly to the UK, where doctors are well paid by a public health system, and where neurologists may be a particularly 'bookish' group. 'Greed' may be hypothesized to be less of an incentive under those conditions, whereas 'guilt' operates consistently across cultures [11].

This survey did not use the full array of features included in the 'Tailored Design Method' [12] or the other techniques shown to increase response rates [13], and it may be that a fully optimised survey would have found different results. Nevertheless, it did use some features, such as first-class stamps, multiple contacts, university sponsorship, and the incentive itself, of course, consistent with what is known about influencing physicians specifically [14].

Finally, any correlations amongst the neurologists' responses in each cluster would mean that we had underestimated the standard errors and would increase our confidence interval for the odds ratio; though the intra-class correlation coefficient of responses was low (0.07), some chance of type 1 error therefore remains since we did not account for clustering in the analysis.

## Conclusions

While pre-payment incentives exert the predicted effect on neurologists' response rates to a survey, post-payment incentives exert no significant effect. This would suggest that any survey of neurologists should avoid incentives contingent on completion as a means of enhancing response. It also suggests that neurologists, in the UK at least, may have a distinct psychology, with promised rewards being a less effective motivator for them than for other physicians.

## Competing interests

The authors declare that they have no competing interests.

## Authors' contributions

RK, SW and DA obtained the funding and designed the study. RK collected the data. RK and DA carried out the statistical analysis. RK wrote the paper, with assistance from SW and DA. All authors read and approved the final manuscript.

## Pre-publication history

The pre-publication history for this paper can be accessed here:

http://www.biomedcentral.com/1471-2377/10/100/prepub
